# Retrospective analysis of histo-pathological patterns in gallbladder diseases

**DOI:** 10.6026/973206300211526

**Published:** 2025-06-30

**Authors:** Shaziya Noor, Namita Kumari

**Affiliations:** 1 Department of Pathology, SKMCH, Muzaffarpur, Bihar, India

**Keywords:** Gall bladder, histopathology, cholecystitis, gall bladder carcinoma, cholecystectomy, adenocarcinoma

## Abstract

A total of 250 gall bladder samples throughout three years at SKMCH Muzaffarpur, India were used. Of all findings chronic cholecystitis proved to be most prevalent (76%) while acute cholecystitis occurred in 8% of cases and xantho-granulomato-uscholecystitis appeared in 4.8% of specimens. We show that 6% of lesions were hyperplastic and neoplastic changes including adenocarcinoma appeared in 5.2% of cases. Incidental gall bladder carcinoma in 1.2% of examined specimens. The study highlights the necessity of routine histopathological examination since it identifies clinically inconspicuous malignancies before proper treatment can start.

## Background:

The majority of gastrointestinal disorders exist from gall bladder diseases which frequently appear in regular surgical care. Right upper quadrant abdominal pain combined with vomiting and nausea represent the predominant symptoms through which patients initially seek medical evaluation because of their cholelithiasis and cholecystitis diagnosis [1]. Healthcare professionals consider cholecystectomy as the preferred treatment for managing most gall bladder diseases in patients who need urgent or scheduled surgery while obtaining important tissue for diagnostic examination. Gallbladder diseases exhibit a wide histopathological spectrum, predominantly inflammatory in nature, emphasizing the critical role of routine histopathological evaluation of cholecystectomy specimens for early detection of pre-malignant and malignant lesions [2]. Gall bladder specimen analysis through histopathological examination serves two essential purposes: it ensures diagnosis accuracy and reveals possible silent premalignant or malignant findings simultaneously [3]. Almost all of the gallbladder lesions are inflammatory in origin, of which the most common disease being chronic cholecystitis in female of 30-40 years presenting with abdominal pain [4]. Gall bladder carcinoma exists as an uncommon malignant disorder which demonstrates an aggressive behavior while presenting a negative prognosis before early detection occurs. The gallbladder is commonly affected by a wide range of pathological conditions, from inflammatory to neoplastic lesions, making its histopathological evaluation essential for accurate diagnosis and effective patient management [5]. Every gall bladder specimen needs routine histopathological examination in order to achieve proper medical diagnosis and appropriate treatment management. The need to understand all possible gall bladder diseases through histological review has intensified because imaging methods demonstrate known limitations. The initial use of ultrasonography as the diagnostic tool does not provide complete assurance for detecting minor mucosal abnormalities and differentiating benign from malignant conditions. Gallbladder diseases encompass a wide range of histopathological conditions, making routine examination of cholecystectomy specimens essential for accurate diagnosis and early detection of potential malignancies [6]. Medical experts report that diagnosis of cholesterolosis and early adenomyomatosis and even carcinoma in situ remains challenging through imaging methods because these conditions can evade detection [7]. Therefore, it is of interest to document the retrospective analysis of histo-pathological patterns in gallbladder diseases.

## Materials and Methods:

The three-year observational research took place at the Pathology Department of SKMCH Muzaffarpur, Bihar, India between September 2020 and August 2023. The department received 250 cholecystectomy specimens during the study period and all these specimens underwent histopathological examination.

## Inclusion criteria:

The research included all gall bladder specimens which underwent cholecystectomy regardless of patient demographic characteristics or missing clinical information.

## Exclusion criteria:

The researchers excluded tissue samples that were insufficient or autolyzed and specimens without necessary clinical data.

## Data collection:

Medical records together with pathology requisition forms supplied patient data about age, sex, presenting complaints, clinical diagnosis, imaging results, laparoscopic or open cholecystectomy procedure type.

## Gross examination:

The specimen needed 10% neutral buffered formalin fixation for 24-48 hours before experts performed thorough gross assessments. The diagnostic assessment included recording observations about external look of the organ, its wall dimensions, gallstone presence and classification, along with mucosal disruption evaluation and extra mass check. Medical staff measured specimen wall thickness with a digital Vernier caliper and classed stones according to their number of pieces alongside dimensions and shape along with color.

## Tissue processing and histological examination:

Multiple representative sections were taken from the neck, body, and fundus of the gall bladder. In cases where gross abnormalities were observed, additional sections were obtained from those regions. The tissues were processed in an automatic tissue processor, embedded in paraffin wax, and sectioned at 4-5 μm thickness. All slides were stained with routine hematoxylin and eosin (H & E) and examined under light microscopy by two independent pathologists.

## Classification of lesions:

Histopathological findings were categorized into:

[1] Inflammatory lesions: acute cholecystitis, chronic cholecystitis, and Xanthogranulomatous cholecystitis.

[2] Hyperplastic changes: adenomyomatosis and cholesterolosis.

[3] Neoplastic lesions: including benign tumors and malignant tumors such as adenocarcinoma.

In cases where malignancy was suspected, additional special stains and histochemical analysis were employed for confirmation. Tumors were graded and staged as per the WHO classification and TNM staging system, wherever applicable.

## Statistical analysis:

Data were compiled using Microsoft Excel and analyzed using descriptive statistics. Results were expressed in terms of frequencies and percentages. Comparative evaluation was also made between clinical diagnosis and histopathological findings to identify any discrepancies.

## Results:

A total of 250 cholecystectomy specimens were analyzed during the study period. The age of patients ranged from 18 to 82 years, with a mean age of 47.6 ± 12.4 years. The majority of patients were in the age group of 41-60 years (44.8%). There was a female predominance, with 182 female patients (72.8%) and 68 male patients (27.2%), giving a female-to-male ratio of approximately 2.7:1. The most common presenting symptoms were right upper quadrant pain (78.4%), dyspepsia (42.0%), nausea/vomiting (38.8%), and jaundice (6.4%). Most patients (88%) underwent laparoscopic cholecystectomy, while the remaining 12% had open surgery due to complications or prior adhesions. Histopathological examination revealed that chronic cholecystitis was the most frequent diagnosis, observed in 190 cases (76%). Acute cholecystitis was seen in 20 cases (8%), followed by Xanthogranulomatous cholecystitis in 12 cases (4.8%). Hyperplastic changes such as cholesterolosis and adenomyomatosis were observed in 15 cases (6%) (Table 1 - see PDF). Neoplastic lesions were noted in 13 cases (5.2%), with adenocarcinoma being the most common, seen in 10 cases (4%) (Table 2 - see PDF).

Incidental gall bladder carcinoma was identified in 3 cases (1.2%) that had no prior clinical or radiological suspicion. A breakdown of gallstone presence revealed that 206 cases (82.4%) had gallstones; with the highest association found in chronic cholecystitis cases (91.6% of chronic cases had stones). The correlation between gallstones and carcinoma was observed in 8 of the 13 neoplastic cases (61.5%) (Table 3 - see PDF). The most frequent incidental finding was adenomyomatosis, followed by cholesterolosis and gall bladder polyps. These findings were not suspected clinically or radiologically in 60% of such cases, indicating the diagnostic value of routine histopathology. As shown in (Tables 1-3 - see PDF), inflammatory lesions dominate the histological spectrum, but clinically silent neoplastic changes and rare variants like carcinosarcoma were also identified, underlining the importance of histopathological assessment.

## Discussion:

Several pathological conditions affect the gall bladder while inflammation stands as the main diagnosis group that also includes hyperplastic and neoplastic changes as occasional findings. The present study revealed chronic cholecystitis as the main diagnosed condition because research around the world identifies it as the primary histological finding in cholecystectomy specimens [1, 2]. The study findings show gallstones as a primary cause of chronic cholecystitis since 91.6% of examined cases exhibited this condition [3, 4]. Areas showing acute cholecystitis developed in 8% of cases whereas other studies mainly detected rates between 10-15% [5].

The reduced prevalence in our study could result from late symptom presentation or treatment of acute conditions in some patients with conservative methods. Xanthogranulomatous cholecystitis appeared as a rare yet aggressive chronic inflammatory condition which represented 4.8% of our studied cases. Previous research shows these frequencies are significant because they can become clinically and radiologically similar to malignant situations [6, 7]. Ultrasound imaging techniques such as contrast-enhanced ultrasound have become essential tools for evaluating gallbladder pathology due to their ability to correlate well with histological findings [8]. Parasitic infections can significantly impact the hepatobiliary and pancreatic systems, necessitating comprehensive diagnostic approaches in both human and veterinary medicine [9]. All patients require histological examination because these mostly benign lesions can appear incidentally during surgical procedures.

Gall bladder carcinoma was identified in 5.2% of specimens, including 1.2% that was incidental findings. This rate aligns with several Indian studies, which have reported an incidence of 3-6% [10, 11]. Adenocarcinoma was the most common histological type, followed by squamous cell carcinoma and rare variants like carcinosarcoma. The female predominance in gall bladder cancer, observed in our study, has been well documented and is attributed to hormonal, genetic and dietary factors [12, 13]. Importantly, a significant proportion of neoplastic lesions in this study were not clinically suspected preoperatively. This finding is in line with reports that emphasize the silent nature of early gall bladder cancers and the limitations of imaging in their detection [14]. Hence, reliance on radiological and gross appearance alone may lead to missed diagnoses. Histopathology remains the gold standard for accurate identification of early malignancies and rare variants [15]. Our study also noted a strong association between gallstones and carcinoma, with 61.5% of cancer cases showing cholelithiasis. Gallstones are considered a major risk factor for gall bladder carcinoma due to chronic mucosal irritation, bile stasis, and increased mucosal turnover [11, 12]. This association further supports the recommendation for routine histopathological examination of all cholecystectomy specimens, even in cases performed for presumed benign conditions.

## Conclusion:

Majority of gall bladder lesions are inflammatory. However, the detection of hyperplastic and malignant changes-some of which are asymptomatic and incidental-justifies the routine microscopic analysis of all gall bladder specimens. Early detection through histopathology can significantly improve patient outcomes, especially in the case of malignancies.
